# Heterogeneous and dynamic lung cancer mortality among immigrants relative to native-born populations in France, 2000–2021

**DOI:** 10.1093/eurpub/ckag134

**Published:** 2026-07-30

**Authors:** Patricia MacNeil, Fanny Godet, Simon Ducarroz, Gwenn Menvielle

**Affiliations:** Université Paris-Saclay, UVSQ, Gustave Roussy, Inserm, CESP, F-94800 Villejuif, France; Université Paris Cité, Inserm, ECEVE, F-750100 Paris, France; CNRS, Institut Convergences Migration, Aubervilliers, France; Inserm, CépiDc, Paris, France; Université Paris Cité, Inserm, ECEVE, F-750100 Paris, France; CNRS, Institut Convergences Migration, Aubervilliers, France; Université Paris-Saclay, UVSQ, Gustave Roussy, Inserm, CESP, F-94800 Villejuif, France

**Keywords:** Immigrants, Migrant health, Lung cancer, Tobacco-related cancer, France

## Abstract

Immigrants tend to have lower lung cancer mortality compared to natives, with some exceptions by region of birth. To date, findings on cancer mortality among immigrants in Europe show that cancer mortality trends vary by host country, region of birth, sex, and cancer type. We aim to describe immigrant lung cancer mortality relative to natives and disparities by region of birth, clarify the role of social factors, and assess temporal trends. We used data from France’s national mortality registry and population censuses to evaluate immigrant and native lung cancer mortality rates for the 2000–2021 period. We estimated sex-specific mortality rate ratios (MRRs) to assess relative lung cancer mortality by region of birth. Interaction was tested and analyses were further stratified by social deprivation and subperiod. Within-region of birth social gradients in lung cancer mortality were assessed. Foreign-born women (MRR: 0.82; 95% CI: 0.75–0.90) and men (MRR: 0.93; 95% CI: 0.88–1.00) had lower lung cancer mortality relative to natives, with some exceptions by region of birth. Interaction and stratified analysis results show that region of birth trends differed across social deprivation groups. Results for within-group social gradients show a divergence between men and women. Differences between immigrants and natives tended to widen over time. Our study adds to our understanding of the complex mechanisms underlying lung cancer mortality among immigrants, and how they diverge from native-born population patterns. These findings highlight questions about how sex, region of birth, and social deprivation intersect in lung cancer mortality.

## Introduction

Lung cancer is the most common cancer among men and second most common among women, representing a large burden of morbidity [1]. Lung cancer is closely associated with immigrant status, with studies showing overall lower lung cancer mortality among immigrants compared to natives [2–4]. However, certain birth country groups show higher mortality rates compared to natives, such as Eastern Europeans in other European countries for lung cancer [2] or Asian males in the USA for tobacco-related cancers [5]. Overall findings on immigrant lung cancer suggest that immigrants are a heterogeneous group that differ both between and within host countries [6].

Smoking is the leading cause of lung cancer, with approximately 80% of cases attributed to tobacco use [7, 8]. The smoking epidemic model describes observed trends in smoking and subsequent mortality, and how these vary by sex and socioeconomic status (SES) [9]. Authors describe a rise in smoking until reaching a peak, followed by a decrease. This is observed first among men then among women to a lesser magnitude, with smoking-related mortality following a similar pattern 20–30 years later. Generally, these phases occur first among the high SES groups followed by the low SES groups [9]. In addition, this epidemic does not occur simultaneously in all regions. Some regions are in the later smoking epidemic stages, such as North America and Europe, while others are in the early stages, such as North Africa and Southeast Asia [10]. Lung cancer mortality is lower in regions in early epidemic stage [9].

Immigrant lung cancer is associated with exposures occurring both pre- and post-migration. In particular, smoking patterns may differ strongly between birth country and host country general populations. As immigrants become more acculturated, their smoking tends to become more similar to smoking in the host country [11]. The theory of social determinants of health highlights the multiple social factors which operate simultaneously to shape individual experiences and ultimately, health outcomes [12]. Using an intersectional lens when exploring lung cancer among immigrants is therefore crucial to provide insights on how sex, SES, migrant status, country of birth, and duration of stay intersect and produce unique outcomes of lung cancer among immigrants [12].

A temporal analysis of this multiplicity of effects is also necessary, given that both smoking prevalence and sociodemographic gradients in smoking evolve over time. This dynamic is captured by the smoking epidemic model [9, 13] and illustrated by the heterogeneous trends in social inequalities in lung cancer mortality across different countries [14]. Data in Spain from 2000 to 2016 revealed that smoking-related cancers surpassed breast cancer as the leading cause of cancer death among immigrant women but not in native women [15].

However, existing studies on lung cancer mortality among immigrants in Europe have been limited in granularity, and have not explored the combined effects of birth country, host country, and time period [2, 16]. Additionally, indicators of SES are included in some studies [6, 16, 17], but not systematically. An intersectional approach to lung cancer mortality among immigrants that accounts for interaction between sociodemographic determinants (i.e. sex, region of birth) and socioeconomic indicators (i.e. social deprivation) is therefore lacking. Our analysis aims are to describe lung cancer mortality among immigrants relative to natives by sex, to examine disparities by region of birth, and to assess temporal trends in these associations. In addition, our analysis seeks to investigate the underlying mechanisms by clarifying the role of social factors. Therefore, we will evaluate social inequalities in lung cancer mortality by region of birth and to what extent social deprivation modifies the association between lung cancer mortality and region of birth. This approach considers social deprivation both as a determinant of lung cancer mortality and as an effect modifier of lung cancer mortality. Results will be discussed within the framework of the smoking epidemic. We hypothesize that we will observe lower lung cancer mortality rates among immigrants from regions in the early stages of the epidemic compared to natives and immigrants from regions in the later stages, such as other European countries. We also hypothesize that social inequalities in lung cancer mortality will differ depending on the stage of the smoking epidemic in the home country.

## Methods

### Data

We obtained exhaustive aggregated mortality data from France’s national death registry (CépiDc) for lung cancer (ICD-10 code C34) mortality by sex, five-year age group, region of birth, year of death, and social deprivation quintile. All data concerns the population aged 30 years and older in Metropolitan France. We analysed mortality data for the 2000–2021 period. Mortality counts for 2011 were not included due to missing region of birth data. Aggregated, anonymous data were used in analyses and therefore did not require ethical approval.

Person years at risk (PYR) were calculated using population census data. In France, since 2004, there is a rolling annual census that covers the entire population over a five-year period. We obtained census data from the National Institute of Statistics and Economic Studies (INSEE) estimated population counts by sex, five-year age group, region of birth, and social deprivation quintile for the following 5 years: 1999, 2006, 2011, 2016, and 2021. Each year of mortality data was assigned the population data of the nearest year.

The French Deprivation Index (FDep) was used as social deprivation indicator [18]. FDep was provided by quintile (1 = least deprived, 5 = most deprived) at the commune level. We used available versions of the FDep from the following years: 1999, 2009, and 2015. We applied the closest FDep version to the population data for each census year. Details on how census, mortality and FDep data were matched throughout the study period are available in Supplementary Table S1.

Data were grouped by region of birth: France (natives); Southern Europe; Other European countries; Maghreb; Other African countries; the Middle East and Turkey; Other Asian countries; and America/Oceania. A full list of countries and corresponding region is provided in Supplementary Table S2. When referring to these groups in the following text, we are referring to individuals born in that region.

### Statistical analysis

We calculated direct age-standardized lung cancer mortality rates per 100 000 (ASMR) using five-year age groups and the 2013 European Standard Population [19]. Lung cancer ASMR were calculated by sex for all foreign-born combined as well as for each region of birth group. In addition, ASMR were also calculated by FDep quintile. Analyses were conducted for the entire study period and by 10-year period. Results were censored for groups where at least one FDep stratum had less than 50 deaths.

Mortality rate ratios (MRR) were calculated using negative binomial regression to model lung cancer mortality among immigrants relative to the native-born group (reference group). The exposure variable was region of birth, assessed first using a binary variable on immigrant status (native-born; foreign-born), then by a region-specific variable. Negative binomial regression was preferred to Poisson regression given the over dispersed nature of the data, and support in the literature that this method is adapted to address severe overdispersion [20]. All models included PYR as an offset term. The MRR were calculated by sex and adjusted on social deprivation and 10-year age group. Within this model, interaction between region of birth and social deprivation quintile and interaction between region of birth and subperiod were tested. Analyses were then stratified by social deprivation quintile. Regression analyses are presented for the entire 2000–2021 period and stratified by 10-year subperiod. Results were censored for groups where at least one FDep stratum had less than 50 deaths.

Finally, we evaluated within-birth country group social inequalities in lung cancer mortality by calculating standardized rate ratios (SRR), which compares each quintile’s ASMR to that of the least deprived group, and the relative index of inequality (RII), which is a comprehensive measure of social inequalities in lung cancer mortality. The RII is calculated using a regression model estimating the linear association between the socioeconomic rank and lung cancer mortality [21]. The rank is a continuous variable that corresponds to the average cumulative frequency of social deprivation, ranging from 0.1 (least deprived quintile) to 0.9 (most deprived quintile). For instance, an RII below 1 indicates decreased mortality with higher deprivation, and above 1 indicates higher mortality in higher deprivation. All models included PYR as an offset term and were adjusted on 10-year age group. RII were calculated using negative binomial regression, or Poisson regression when there was no overdispersion present. Analyses were conducted among native-born, among all foreign-born combined and by country of birth, for the entire 2000–2021 period and by subperiod.

## Results

Compared to natives, for the entire 2000–2021 period, lung cancer mortality was lower among all immigrant women (MRR: 0.82; 95% CI: 0.75–0.90) and men (MRR: 0.93; 95% CI: 0.88–1.00) (Table 1). Among women, lower mortality was observed in all regions of birth except for other European countries. Among men, the mortality did not differ from natives among other European or Maghrebin groups. Southern European men had a higher relative rate (MRR: 1.07; 1.00–1.15). Sex differences in ASMR varied according to region of birth. For example, among the native-born group, the ASMR for men was almost four times higher than that for women (ASMR: 122.4; 95% CI: 122.0–122.8 and ASMR: 33.9; 95% CI: 33.7–34.1, respectively), whereas in the Asian groups, the ASMR for men was approximately twice that of women (ASMR: 65.7; 95% CI: 62.5–69.0 and ASMR: 32.2; 95% CI: 30.3–34.3, respectively).

**Table 1.** Sex-specific population counts, deaths, age-standardized mortality rates (ASMR) per 100 000 and 95% confidence intervals, and mortality rate ratios and 95% confidence intervals for lung cancer deaths among over 30 year olds in France for 2000–2021, 2000–2010 and 2012–2021.^a^

	Women						Men					
Region of birth		PYR	Deaths	Age-standardized mortality rate (95% CI)	Mortality rate ratio (95% CI)	*P*	PYR		Deaths	Age-standardized mortality rate (95% CI)	Mortality rate ratio (95% CI)	*P*
2000–2021												
France		385 120 993	137 989	33.9 (33.7–34.1)	1 (ref)		339 404 596		388 773	122.4 (122.0–122.8)	1 (ref)	
All foreign-born		62 978 083	17 723	28.6 (28.1–29.0)	0.82 (0.75–0.90)		59 718 976		64 077	114.5 (113.6–115.4)	0.93 (0.88–1.00)	
Southern Europe		12 253 319	3889	23.4 (22.7–24.2)	0.70 (0.62–0.78)		11 515 907		18 206	130.0 (128.1–131.9)	1.07 (1.00–1.15)	
Other European		10 390 414	4518	42.1 (40.9–43.4)	1.10 (0.98–1.23)		7 843 624		8972	118.5 (116.0–121.0)	0.96 (0.90–1.03)	
Maghreb		23 478 722	6294	25.5 (24.9–26.2)	0.70 (0.62–0.78)		24 504 584		30 155	117.9 (116.5–119.2)	0.97 (0.91–1.04)	
Sub-Saharan Africa		7 346 193	1122	29.7 (27.8–31.7)	0.77 (0.67–0.87)	*^b^	7 253 761		2672	63.6 (60.6–66.6)	0.54 (0.50–0.59)	*^b^
Turkey and Middle East		2 384 186	452	34.1 (30.9–37.6)	0.86 (0.74–0.99)	*^b^	2 881 653		1771	117.3 (111.3–123.6)	0.88 (0.81–0.96)	
Asia		4 942 355	1056	32.2 (30.3–34.3)	0.87 (0.77–0.99)	*^c^	4 202 964		1818	65.7 (62.5–69.0)	0.54 (0.49–0.59)	*^b^
Oceania/America		2 182 893	392	32.9 (29.4–36.6)	0.84 (0.72–0.98)		1 516 484		483	60.2 (54.5–66.3)	0.48 (0.43–0.54)	
2000–2010												
France		19 488 7132	56557	28.3 (28.1–28.5)	1 (ref)		171 134 754		202 307	132.9 (132.3–133.5)	1 (ref)	
All foreign-born		30 011 531	7653	26.7 (26.0–27.2)	0.90 (0.82–0.99)		2 904 5770		33 892	133.2 (131.7–134.6)	0.99 (0.91–1.06)	
Southern Europe		6 723 536	1771	20.7 (19.8–21.8)	0.71 (0.63–0.83)		6 304 537		10 267	144.8 (142.0–147.7)	1.06 (0.98–1.15)	
Other European		4 883 230	2087	40.0 (38.2–41.8)	1.26 (1.12–1.41)		3 611 464		4837	136.9 (133.0–140.8)	1.00 (0.92–1.08)	
Maghreb		11 480 371	2544	22.7 (21.9–23.7)	0.74 (0.66–0.83)		12 291 558		15 708	133.7 (131.5–135.9)	0.99 (0.92–1.07)	
Sub-Saharan Africa		2 891 858	402	31.4 (27.9–35.2)	0.92 (0.79–1.06)		3 033 488		1199	85.2 (79.0–91.8)	0.62 (0.56–0.69)	
Turkey and Middle East		10 453 328	217	39.5 (34.1–45.4)	1.13 (0.94–1.34)		1 296 789		763	128.3 (118.0–139.2)	0.90 (0.80–1.00)	
Asia		2 114 317	491	37.6 (34.2–41.2)	1.15 (0.99–1.32)		1 882 969		889	85.6 (79.6–92.0)	0.61 (0.55–0.68)	
Oceania/America		872 889	141	31.0 (25.6–37.1)	0.91 (0.74–1.12)		624 965		229	72.3 (62.5–83.2)	0.53 (0.46–0.62)	
2012–2021												
France		190 233 861	81432	38.9 (38.6–39.2)	1 (ref)		168 269 843		186 466	111.8 (111.3–112.3)	1 (ref)	
All foreign-born		32 966 553	10070	30.0 (29.4–30.6)	0.77 (0.69–0.85)		30 673 205		30 185	99.0 (97.9–100.1)	0.90 (0.85–0.96)	
Southern Europe		5 529 783	2118	26.3 (25.1–27.5)	0.69 (0.61–0.79)		5 211 369		7939	114.3 (111.8–116.9)	1.07 (1.00–1.14)	
Other European		5 507 185	2431	43.9 (42.2–45.7)	1.03 (0.92–1.17)		4 232 159		4135	101.8 (98.7–105.0)	0.93 (0.87–1.00)	
Maghreb		11 998 351	3750	27.4 (26.6–28.3)	0.67 (0.59–0.75)		12 213 026		14 447	104.4 (102.7–106.1)	0.95 (0.90–1.01)	
Sub-Saharan Africa		4 454 335	720	28.5 (26.1–30.9)	0.68 (0.59–0.79)		4 220 273		1473	53.3 (50.1–56.6)	0.52 (0.48–0.57)	
Turkey and Middle East		1 338 859	235	30.2 (26.2–34.5)	0.69 (0.58–0.82)		1 584 863		1008	109.4 (102.0–117.1)	0.91 (0.83–0.99)	
Asia		2 828 037	565	28.3 (25.9–30.8)	0.71 (0.61–0.82)		2 319 995		929	53.2 (49.6–57.0)	0.50 (0.46–0.55)	
Oceania/America		1 310 003	251	33.8 (29.4–38.7)	0.78 (0.65–0.94)		891 519		254	53.0 (46.0–60.7)	0.45 (0.39–0.52)	

a Models adjusted on region of birth, FDep (in quintiles), and 10-year age group. *P* indicates significance of interaction between region of birth (ref = France) and subperiod (ref = 2000–2010).

b *P* < .05.

c *P* < .001.

Analyses by subperiod showed that differences in mortality between natives and all foreign-born increased between the 2000–2010 and 2012–2021 subperiods both for women (MRR: 0.90; 95% CI: 0.82–0.99 and MRR: 0.77; 95% CI: 0.69–0.85, respectively) and men (MRR: 0.99; 95% CI: 0.91–1.06 and MRR: 0.90; 95% CI: 0.85–0.96, respectively), although confidence intervals overlapped. This change was driven by an increase in female native-born mortality over time (ASMR: 28.3; 95% CI: 28.1–28.5 and ASMR: 38.9; 95% CI 38.6–39.2) contrasted with a decreasing trend in mortality rate among Sub-Saharan African (interaction *P* < .05), Asian (interaction *P* < .001), Turkish and Middle Eastern (interaction *P* < .05) women. Conversely, mortality in native men decreased (ASMR: 132.9; 95% CI: 132.3–133.5 and ASMR: 111.8; 95% CI: 111.3–112.3), but more slowly than among foreign-born groups. Interaction results further suggest decreasing relative mortality among sub-Saharan African and Asian men (interaction *P* < .05). Multivariable results remained largely similar when adjusted on 5-year age groups instead of 10-year age groups, as shown in Supplementary Table S3.

Table 2 displays how immigrant lung cancer mortality relative to natives varies across deprivation quintiles. Among women, the same mortality pattern was observed across deprivation quintiles except in the Turkish and Middle Eastern group, where the MRR was higher in the lowest deprivation quintile (1.30; 95% CI: 1.03–1.63) and lower in the three higher deprivation groups compared to their native counterparts (test for interaction *P* < .05 for *Q*3 and *P* < .001 for *Q*4 and *Q*5). Among men, variation in relative mortality across deprivation groups was observed in the Maghrebin group, where the MRR was higher in the lowest deprivation group (1.08; 95% CI: 1.00–1.17) and lower in the highest deprivation group (0.85; 95% CI: 0.72–1.00) compared to their native counterparts (*P* < .05 for interaction at *Q*5). Additionally, the MRR in Turkish and Middle Eastern men was lower in the highest deprivation group (*P* < .05 for interaction at *Q*4 and *Q*5), and not different in the other deprivation groups compared to native counterparts. Finally, the gradient was negative but more pronounced among men in high deprivation groups in Asia (*P* < .05 for interaction at *Q*5) and Sub-Saharan (*P* < .001 for interaction at *Q*5) men. These results are consistent across 10-year subperiods, as shown in Supplementary Tables S4a and b.

**Table 2.** Age-standardized mortality rates (ASMR) per 100 000 and mortality rate ratios of lung cancer by social deprivation, sex, and region of birth for 2000–2021.^a^^,^^b^

	Quintile 1 (lowest deprivation)			Quintile 2		Quintile 3		Quintile 4		Quintile 5 (highest deprivation)			*P*
Region of birth	ASMR (95% CI)	MRR (95% CI)		ASMR (95% CI)	MRR (95% CI)	ASMR (95% CI)	MRR (95% CI)	ASMR (95% CI)	MRR (95% CI)	ASMR (95% CI)		MRR (95% CI)	
Women													
France	33.6 (33.1–34.0)	1 (ref)		32.0 (31.6–32.4)	1 (ref)	32.6 (32.2–33.0)	1 (ref)	32.5 (32.1–32.9)	1 (ref)	33.5 (33.1–33.9)		1 (ref)	
All foreign-born	28.3 (27.4–29.2)	0.84 (0.74–0.96)		27.1 (26.2–28.1)	0.87 (0.74–1.01)	27.2 (26.3–28.2)	0.81 (0.70–0.94)	28.4 (27.4–29.4)	0.83 (0.69–1.00)	25.5 (24.7–26.5)		0.77 (0.60–0.97)	
Southern Europe	22.0 (20.4–23.7)	0.67 (0.56–0.80)		22.5 (20.9–24.3)	0.74 (0.59–0.92)	23.0 (21.3–24.9)	0.70 (0.58–0.83)	23.3 (21.5–25.2)	0.72 (0.57–0.93)	22.2 (20.6–24.0)		0.68 (0.53–0.89)	
Other European	37.0 (34.7–39.4)	1.03 (0.86–1.22)		38.5 (35.7–41.6)	1.15 (0.93–1.42)	40.1 (37.2–43.0)	1.14 (0.96–1.36)	42.9 (40.0–45.9)	1.13 (0.89–1.43)	43.6 (40.7–46.5)		1.14 (0.89–1.47)	
Maghreb	25.6 (24.2–27.0)	0.76 (0.64–0.90)		24.8 (23.5–26.3)	0.77 (0.62–0.94)	25.1 (23.7–26.5)	0.73 (0.62–0.86)	25.1 (23.6–26.7)	0.69 (0.54–0.87)	21.2 (19.9–22.6)		0.59 (0.46–0.76)	*^A^
Sub-Saharan Africa	31.1 (27.4–35.1)	0.84 (0.69–1.03)		30.1 (25.9–34.8)	0.87 (0.69–1.11)	25.6 (21.6–30.2)	0.71 (0.57–0.88)	30.1 (25.3–35.5)	0.83 (0.63–1.09)	23.9 (19.5–28.9)		0.62 (0.46–0.83)	
Turkey and Middle East	46.0 (38.8–54.1)	1.30 (1.03–1.63)		40.6 (31.9–50.8)	1.16 (0.86–1.56)	26.3 (19.7–34.2)	0.69 (0.51–0.93)	23.8 (17.5–31.4)	0.58 (0.41–0.82)	23.8 (17.9–30.7)		0.58 (0.41–0.81)	*^B^
Asia	31.3 (28.0–34.8)	0.9 (0.74–1.09)		28.5 (24.5–33.0)	0.84 (0.65–1.07)	29.0 (24.5–34.1)	0.80 (0.63–1.00)	37.5 (31.7–44.0)	0.99 (0.74–1.31)	33.2 (28.0–39.0)		0.89 (0.66–1.19)	
Oceania/America	30.4 (25.3–36.1)	_		30.3 (23.0–39.2)	–	33.6 (25.0–44.0)	–	37.7 (28.0–49.6)	–	26.4 (18.0–37.1)		–	
Men													
France	99.8 (98.2–100.6)	1 (ref)		110.7 (109.9–111.6)	1 (ref)	117.5 (116.6–118.3)	1 (ref)	123.0 (122.2–123.9)	1 (ref)	138.8 (137.9–139.7)		1 (ref)	
All foreign-born	95.9 (94.2–97.7)	0.96 (0.92–1.01)		107.0 (105.0–109.0)	0.97 (0.87–1.07)	110.8 (108.8–112.8)	0.92 (0.83–1.03)	118.7 (116.6–120.9)	0.95 (0.83–1.08)	120.0 (118.0–122.0)		0.86 (0.71–1.04)	
Southern Europe	107.4 (103.5–111.4)	1.09 (1.00–1.19)		118.4 (114.4–122.5)	1.09 (0.97–1.22)	123.3 (119.1–127.6)	1.05 (0.92–1.20)	135.1 (130.8–139.6)	1.09 (0.97–1.24)	140.3 (136.0–144.8)		1.03 (0.87–1.23)	
Other European	96.0 (91.5–100.7)	0.97 (0.89–1.06)		109.5 (103.8–115.5)	0.99 (0.88–1.12)	113.5 (108.0–119.3)	0.95 (0.83–1.09)	116.2 (110.9–121.8)	0.94 (0.82–1.07)	135.2 (129.6–141.1)		0.96 (0.81–1.14)	
Maghreb	104.5 (101.8–107.3)	1.08 (1.00–1.17)		110.9 (107.9–113.9)	1.00 (0.90–1.12)	111.8 (108.9–114.8)	0.94 (0.83–1.07)	120.8 (117.6–124.2)	0.98 (0.87–1.10)	119.0 (116.1–122.0)		0.85 (0.72–1.00)	*^C^
Sub-Saharan Africa	60.7 (55.5–66.1)	0.65 (0.58–0.72)		58.3 (52.2–64.8)	0.58 (0.50–0.67)	66.4 (59.3–74.0)	0.56 (0.47–0.65)	74.1 (65.4–83.5)	0.55 (0.47–0.64)	56.7 (50.3–63.5)		0.39 (0.32–0.47)	*^D^
Turkey and Middle East	108.3 (97.1–120.3)	1.06 (0.93–1.20)		118.4 (103.2–135.1)	0.97 (0.82–1.14)	114.9 (100.4–130.6)	0.87 (0.73–1.03)	106.7 (93.1–121.6)	0.78 (0.66–0.92)	119.2 (106.1–133.2)		0.75 (0.62–0.92)	*^E^
Asia	56.5 (51.4–61.8)	0.57 (0.51–0.64)		66.7 (59.7–74.1)	0.59 (0.51–0.69)	75.0 (66.5–84.2)	0.58 (0.49–0.69)	68.1 (59.3–77.6)	0.52 (0.44–0.62)	64.9 (56.6–73.9)		0.41 (0.33–0.51)	*^F^
Oceania/America	49.3 (41.4–58.1)	0.47 (0.40–0.56)		54.0 (42.0–68.2)	0.48 (0.38–0.61)	66.6 (51.9–84.0)	0.52 (0.40–0.67)	68.0 (51.9–87.2)	0.52 (0.40–0.68)	70.5 (52.7–91.9)		0.42 (0.31–0.57)	

a Models adjusted on region of birth and 10-year age group. Cells marked “–” indicate censored region of birth groups due to low death count.

b *P* indicates significance of interaction between region of birth (ref = France) and social deprivation quintile (ref = *Q*1) for the following quintiles: ^A^*Q*5 (*P* = .051), ^B^*Q*3 (*P* < .05), *Q*4 and *Q*5 (*P* < .001), ^C^*Q*5 (*P* <0.05), ^D^*Q*5 (*P* < .001), ^E^*Q*4 and *Q*5 (*P* < .05), ^F^*Q*5 (*P* < .05).

Table 3 shows sex and region of birth-specific SRR and RII for the entire 2000–2021 period and subperiods 2000–2010 and 2012–2021, displaying social gradients of lung cancer mortality by sex and region of birth. Among women, no association between social deprivation and lung cancer mortality was observed among natives or the Southern European group for the entire period. By contrast, mortality decreased as deprivation increased in Sub-Saharan African (RII: 0.79; 95% CI: 0.62–1.00), Maghrebin (RII: 0.83; 95% CI: 0.75–0.91), and Turkish and Middle Eastern women (RII: 0.38; 95% CI: 0.28–0.53). These results were consistent in both subperiods for the latter two groups. The same was observed among all foreign-born women in 2000–2010 (RII: 0.87; 95% CI: 0.79–0.97). On the other hand, other European women showed a gradient indicating higher mortality among high deprivation groups, but only in the most recent subperiod (RII: 1.42; 95% CI: 1.12–1.80). The same trend emerged in the last subperiod among native women but was non-significant (RII: 1.17; 95% CI: 0.99–1.39).

**Table 3.** Standardized rate ratios (SRR) of lung cancer by sex and region of birth for 2000–2021 and relative indices of inequality (RII) for 2000–2021, 2000–2010 and 2012–2021.^a^

	**SRR**					RII (95% CI)		
	2000–2021					2000–2021	2000–2010	2012–2021
Region of birth	*Q*1/*Q*1	*Q*2/*Q*1	*Q*3/*Q*1	*Q*4/*Q*1	*Q*5/*Q*1			
Women								
France	1	0.95	0.97	0.97	1	1.06 (0.92, 1.23)	0.92 (0.82–1.05)	1.17 (0.99–1.39)
All foreign-born	1	0.96	0.96	1	0.9	0.92 (0.84–1.01)	0.87 (0.79–0.97)	0.97 (0.86–1.11)
Southern Europe	1	1.02	1.05	1.06	1.01	0.99 (0.88, 1.10)	1.00 (0.84–1.18)	0.98 (0.84–1.15)
Other European	1	1.04	1.08	1.16	1.18	1.18 (0.97, 1.42)	0.97 (0.80–1.16)	1.42 (1.12–1.80)
Maghreb	1	0.97	0.98	0.98	0.83	0.83 (0.75, 0.91)	0.76 (0.64–0.90)	0.87 (0.76–1.00)
Sub-Saharan Africa	1	0.97	0.82	0.97	0.77	0.79 (0.62, 1.00)	0.82 (0.58–1.16)	0.78 (0.59–1.03)
Turkey and Middle East	1	0.88	0.57	0.52	0.52	0.38 (0.28, 0.53)	0.52 (0.32–0.82)	0.31 (0.20–0.49)
Asia	1	0.91	0.93	1.2	1.06	1.13 (0.87, 1.46)	1.30 (0.96–1.78)	1.00 (0.74–1.33)
Oceania/America	1	1	1.11	1.24	0.87	1.07 (0.91, 1.24)	1.31 (0.74–2.35)	0.95 (0.61–1.48)
Men								
France	1	1.11	1.18	1.23	1.39	1.53 (1.36, 1.72)	1.46 (1.31–1.63)	1.60 (1.40–1.83)
All foreign-born	1	1.12	1.16	1.24	1.25	1.33 (1.28–1.39)	1.36 (1.28–1.45)	1.33 (1.26–1.39)
Southern Europe	1	1.1	1.15	1.26	1.31	1.43 (1.33, 1.54)	1.42 (1.29–1.55)	1.39 (1.28–1.50)
Other European	1	1.14	1.18	1.21	1.41	1.48 (1.35, 1.62)	1.50 (1.33–1.68)	1.46 (1.31–1.64)
Maghreb	1	1.06	1.07	1.16	1.14	1.19 (1.14, 1.25)	1.21 (1.14–1.30)	1.21 (1.13–1.30)
Sub-Saharan Africa	1	0.96	1.09	1.22	0.93	0.93 (0.79, 1.10)	0.93 (0.77–1.13)	0.99 (0.80–1.24)
Turkey and Middle East	1	1.09	1.06	0.99	1.1	1.06 (0.91, 1.24)	1.05 (0.82–1.33)	1.11 (0.90–1.38)
Asia	1	1.18	1.33	1.21	1.15	1.18 (1.01, 1.39)	1.44 (1.14–1.82)	1.05 (0.85–1.31)
Oceania/America	1	1.1	1.35	1.38	1.43	1.53 (1.12–2.07)	1.42 (0.91–2.23)	1.72 (1.13–2.62)

a Models adjusted on region of birth and 10-year age group.

Among men, we observed a social gradient in most region groups, where mortality increased as deprivation increased. This gradient was observed for French, European-born, and Oceania/America-born men, and to a lesser extent, Maghrebin and Asian men. These results were consistent across subperiods, with the exception of Asian men where inequalities were observed only in the most recent subperiod.

## Discussion

This ecological study found overall lower lung cancer mortality among both female and male immigrants in France over a 20-year period. Results revealed that differences between immigrants and natives widened over time, with relative mortality decreasing among female and male immigrants contrasted with an increase in female native-born mortality and a slower decrease in male native-born mortality. However, some region of birth groups deviated from this trend, such as other European women who became more similar to French-born women, and Southern European men who surpassed native-born lung cancer over time. Additionally, we have clarified the role of social deprivation and highlighted intersectional effects. In particular, we observed differences in relative lung cancer mortality by region of birth across deprivation quintiles as well as in social gradients in lung cancer mortality by region of birth, with none, positive or negative associations.

Our results on lower relative immigrant lung cancer mortality are consistent with previous findings [2, 4, 16]. While this study did not measure individual smoking history, it is important to look to broad regional trends in smoking to contextualize our empirical findings and discuss the results within the framework of the smoking epidemic. These results are consistent with differences in past smoking rates between regions of birth in particular, in 1970 when North American and European smoking rates were much higher than smoking rates in most African countries [10]. In other words, our findings on lung cancer mortality are consistent with differences in smoking epidemic stage advancement between regions [10]. In addition, studies of immigrant health outcomes require a life course perspective, accounting for exposures both pre- and post-migration. In the case of immigrants arriving in a high-income country (HIC) from low- and middle-income countries (LMIC), regional differences in smoking epidemic context should be considered. In the context of several LMIC, the original smoking epidemic model remains informative for men but does not apply to women, whose smoking and related mortality rates have remained very low, such as in China and India [13]. Overall, many LMIC are in the early phases of the epidemic, with high smoking rates among men [10]. In addition, the influence of birth country may decrease over time particularly when the time spent in the host country (i.e. France) surpasses the lung cancer latency period of several decades [9]. There is evidence that immigrant smoking in HIC tends to become more similar to native population smoking with increased duration of stay [11]. Therefore, it is important to distinguish between immigrant groups who have been present for several decades, and those who have migrated more recently. To illustrate, in France, immigrants from Sub-Saharan Africa are more recently established compared to Maghrebin immigrants, representing 24% of all African immigrants in 1999, compared to 40% in 2023 [22]. Our findings on relative mortality in Sub-Saharan African groups align with the expectation that newly arrived immigrants from LMIC, who generally have lower past smoking exposure, exhibit lower lung cancer mortality than earlier-arrived migrants from LMIC (e.g. Turkey and Middle East, Maghreb) when compared to natives.

Regarding trends over the 20-year period, more exploration is needed to understand why the immigrant-native mortality gap widened over time, and why we observe different trajectories between regions of birth in lung cancer mortality.

Additionally, we have clarified the role of social deprivation in lung cancer mortality by region of birth and highlighted intersectional effects. We identified variations in the social gradients of lung cancer mortality by sex and region of birth. These differences can be interpreted in light of the stages of smoking epidemic stages [9]. In particular, variation in social gradients, especially among women, may reflect the stage of the smoking epidemic in the home country smoking epidemic stage. First, among French-born women, no significant gradient was observed, consistent with the rapid inversion of educational gradients in smoking several decades ago in France showing that French women reached the last stage of the epidemic [23]. Over time, the educational inequalities observed in smoking among later birth cohorts of French-born women will likely emerge in lung cancer mortality and already appears in the most recent subperiod. Second, our results for European-born women are consistent with findings on socioeconomic inequalities in lung cancer, showing that Northern and Eastern European woman are at a more advanced stage of the smoking epidemic that Southern European women [14]. Third, women born in Sub-Saharan Africa, Turkey, and the Middle East and Asia displayed higher mortality in low deprivation groups. Diverging gradients between native-born and Sub-Saharan African-born women may be partly explained by regional differences in past social gradients in smoking. However, this comparison cannot be made definitively as there is little evidence on socioeconomic gradients in smoking among Sub-Saharan African populations 20–30 years ago, and smoking has broadly remained low [24]. Moreover, social gradients of smoking may not evolve in the same way in LMIC contexts as in HIC contexts. Current evidence shows that unlike in HIC, smoking adoption did not diffuse from high to low SES groups, possibly because high SES individuals did not smoke in large numbers due to greater awareness of health risks [24]. More precisely, at the individual level, smoking in LMIC is associated with low education level [25].

Among men, strong social gradients indicating high mortality in high deprivation groups were present in the French, European, and America/Oceania-born groups, and to a lesser extent in the Maghrebin and Asian-born groups. Results for the French and European-born groups are consistent with past educational inequalities in smoking [14, 23] and more recent educational inequalities in lung cancer mortality [14].

In addition to assessing social gradients in lung cancer mortality by region of birth, our study provided, to our knowledge, the first evaluation of the extent to which social deprivation modifies the association between lung cancer mortality and region of birth. These findings further underscore the importance of considering multiple social determinants at once, such as sex, region of birth, and deprivation to precisely understand the underlying mechanisms at play. Indeed, we found that deprivation acted as an effect modifier for the association between region of birth and lung cancer for Turkish and Middle Eastern women and for Maghrebin men. While not evaluated in this analysis, we hypothesize that this finding may be linked to acculturation factors. The mortality advantage observed among migrants in some high deprivation groups may reflect a higher number of recently arrived immigrants, who tended to have more favourable health risk behaviours compared to immigrants with a long duration of stay, who are more likely to have adopted the risk profile of native-born population [26]. Additionally, evidence from the USA showed that among some ethnic minority and immigrant groups, low SES was associated with higher levels of familial support compared to high SES [27]. This may in turn influence health behaviours and help prevent the adoption of risk behaviours prevalent in the host country. Future research is needed to examine whether the observed effect modification is attributable to variations in acculturation processes across SES strata among certain region of birth groups.

Selection effects, and in particular the Healthy Migrant Effect should also be considered when investigating migrant health. This refers to the observed health or mortality advantage among immigrants compared to the native-born population [28]. In our study, European immigrants (excluding Southern European females) diverged from the lower relative lung cancer mortality trend, consistent with higher relative lung and bronchus cancer mortality observed in Eastern European immigrants in other European countries [2]. Authors hypothesized that the healthy migrant effect may be less relevant for European immigrants in Europe due to close proximity and free labour movement within the European Union (EU), potentially leading to less selectivity compared to other regions of birth [2]. Although this study only included Eastern European immigrants, the same explanation likely applies to Southern Europeans [2].

Several important lung cancer risk factors could also account for our findings but were not available for our analysis. In addition to smoking, occupational exposures to other carcinogens, second-hand smoke, and environmental pollution are also risk factors for lung cancer [29]. In France, air pollution was found to increase with area-level deprivation [30], which may disproportionally affect immigrants, who are over represented in the high deprivation groups [31]. Similarly, immigrants are also overrepresented among unskilled workers [32], who have higher exposure to occupational carcinogens in France [33]. Additionally, immigrants have a lower use of healthcare and health prevention services in France [34], potentially leading to a later diagnosis and higher mortality risk. Overall, we would expect these inequalities to disproportionately increase lung cancer risk among immigrants compared to natives. Consequently, lung cancer mortality among immigrants might be even lower in the absence of non-smoking-related exposures, although these exposures contribute a relatively smaller proportion to overall lung cancer risk compared to smoking.

### Methodological aspects

This study used exhaustive national mortality data for a 20-year period which permitted the analysis of lung cancer mortality trends among immigrants and natives over time while distinguishing between birth regions. However, as for other studies on immigrant mortality, the Salmon bias may have led to an underestimation of immigrant mortality due in particular to return migration following retirement or illness [28]. This would imply that real differences between immigrant and native mortality would be less pronounced. Although, a study found that return migration only partly accounts for the migrant mortality advantage [35].

As stated, several individual factors were lacking from analysis, limiting the interpretation of the findings. This includes individual smoking history, duration of stay in France, individual SES indicators, and consideration of lung cancer risk factors other than smoking.

We must also consider the historical context of migration in France. The Maghrebin-born group in this study includes repatriates from Algeria, referring to French nationals who moved to metropolitan France following Algeria’s independence in 1962, when it ceased to be a French colony [22]. Their sociodemographic profiles likely resemble those of the native-born population more closely than those of individuals born in the Maghreb without French nationality [36]. Beyond this specific situation, in 2021, 1.7 million people in France were born abroad with French nationality [36]. In future studies, nationality at birth, which is not currently available in France’s mortality registry, may be included to clarify this aspect.

We used a social deprivation index (the FDep), as a measure of SES. At the national level, the FDep has effectively captured deprivation in both urban and rural settings [18]. Nonetheless, we used this index at the commune level, which may have resulted in smaller inequalities compared to an index at a smaller geographical area [37]. Ideally, individual-level indicators would be used to evaluate several of the proposed pathways between SES and health [38]. Future research may seek to understand how SES changes over the immigrant life course and how this impacts social gradients in health outcomes.

Our study adds to our understanding of the complex mechanisms underlying lung cancer mortality among immigrants, and how these differ from patterns observed in native-born populations. Moreover, our analyses raise several questions concerning how sex, region of birth, and social deprivation intersect in patterns of lung cancer mortality. These findings underscore the heterogeneous and dynamic nature of immigrant populations, and clarify their position relative to native lung cancer mortality outcomes. In order to adequately address migrant population health needs, their health outcomes and associated determinants must first be described. In contexts where migrant health is politicized, population-based studies bring us closer to an accurate understanding of what health-related challenges immigrant populations face as they change over time.

## Supplementary Material

ckag134_Supplementary_Data

## Data Availability

Mortality counts by region of birth, sex, age, year, and deprivation quintile can be accessed through formal request to France’s Epidemiology Center for Medical Causes of Deaths (CépiDc) by researchers who meet the criteria for accessing data. Population counts by region of birth, sex, age, year, and deprivation quintile can be accessed through formal request to France’s National Institute of Statistics and Economic Studies (Insee) by researchers who meet the criteria for accessing data. Key points Immigrants tended to have lower lung cancer mortality than native-born groups. Relative lung cancer mortality varied in some region of birth groups by social deprivation. Within-group social gradients in lung cancer mortality varied by region of birth. These findings highlight the dynamic nature of immigrant populations, and raise questions about how sex, region of birth, and social deprivation intersect in lung cancer mortality.
