# Supplementary material for: Heterogeneous and dynamic lung cancer mortality among immigrants relative to native-born populations in France, 2000–2021
Source: Eur J Public Health. 2026 Jul 30;36(4):ckag134. doi: 10.1093/eurpub/ckag134 (PMC13424438; doi:10.1093/eurpub/ckag134)
Supplement: ckag134_Supplementary_Data [file ckag134_supplementary_data.zip › ejph-2026-01-om-0092-File004.docx]

| **Supplementary table 3. Sex-specific and mortality rate ratios and 95% confidence intervals for lung cancer deaths among >30 year olds in France for 2000-2010 and 2010-2021 and 2000-2021.** | | | | | | | | | |
| --- | --- | --- | --- | --- | --- | --- | --- | --- | --- |
|  |  | **Mortality rate ratio (95% CI)** | | | | | | |  |
| **Women** | | | | | | | | | |
| **Region of birth** |  | **2000-2010** |  | **2012-2021** |  | **2000-2021** | |  |  |
| **France** |  | 1 (ref) |  | 1 (ref) |  | 1 (ref) | |  |  |
| **All foreign-born** |  | 0.89 (0.83-0.95) |  | 0.75 (0.69-0.80) |  | 0.80 (0.75-0.86) | |  |  |
| **Southern Europe** |  | 0.69 (0.63-0.76) |  | 0.67 (0.60-0.74) |  | 0.67 (0.61-0.73) | |  |  |
| **Other European** |  | 1.27 (1.16-1.39) |  | 1.03 (0.93-1.13) |  | 1.09 (1.00-1.19) | |  |  |
| **Maghreb** |  | 0.74 (0.68-0.81) |  | 0.66 (0.60-0.72) |  | 0.69 (0.63-0.75) | |  |  |
| **Sub-Saharan Africa** |  | 0.92 (0.81-1.05) |  | 0.68 (0.61-0.77) |  | 0.77 (0.70-0.85) | |  |  |
| **Türkiye and Middle East** |  | 1.13 (0.96-1.32) |  | 0.68 (0.58-0.79) |  | 0.84 (0.74-0.96) | |  |  |
| **Asia** |  | 1.14 (1.01-1.29) |  | 0.69 (0.61-0.78) |  | 0.86 (0.77-0.96) | |  |  |
| **Oceania/America** |  | 0.91 (0.76-1.10) |  | 0.77 (0.66-0.90) |  | 0.83 (0.73-0.95) | |  |  |
| **Men** | | | | | | | | | |
| **Region of birth** |  | **2000-2010** |  | **2012-2021** |  | **2000-2021** |  |  |  |
| **France** |  | 1 (ref) |  | 1 (ref) |  | 1 (ref) |  |  |  |
| **All foreign-born** |  | 0.97 (0.91-1.02) |  | 0.89 (0.85-0.93) |  | 0.92 (0.87-0.96) |  |  |  |
| **Southern Europe** |  | 1.03 (0.97-1.10) |  | 1.05 (0.99-1.10) |  | 1.05 (0.99-1.10) |  |  |  |
| **Other European** |  | 0.98 (0.92-1.05) |  | 0.92 (0.87-0.97) |  | 0.94 (0.89-1.00) |  |  |  |
| **Maghreb** |  | 0.98 (0.92-1.04) |  | 0.94 (0.89-0.98) |  | 0.95 (0.91-1.00) |  |  |  |
| **Sub-Saharan Africa** |  | 0.63 (0.58-0.68) |  | 0.52 (0.49-0.56) |  | 0.54 (0.51-0.58) |  |  |  |
| **Türkiye and Middle East** |  | 0.89 (0.81-0.98) |  | 0.91 (0.84-0.99) |  | 0.88 (0.82-0.94) |  |  |  |
| **Asia** |  | 0.61 (0.55-0.66) |  | 0.50 (0.46-0.54) |  | 0.54 (0.50-0.57) |  |  |  |
| **Oceania/America** |  | 0.53 (0.46-0.62) |  | 0.45 (0.39-0.52) |  | 0.48 (0.43-0.53) |  |  |  |
| **Note:** Models adjusted on region of birth, FDep (in quintiles) and 5-year age group. | | | | | | | | | |
