# Supplementary material for: Heterogeneous and dynamic lung cancer mortality among immigrants relative to native-born populations in France, 2000–2021
Source: Eur J Public Health. 2026 Jul 30;36(4):ckag134. doi: 10.1093/eurpub/ckag134 (PMC13424438; doi:10.1093/eurpub/ckag134)
Supplement: ckag134_Supplementary_Data [file ckag134_supplementary_data.zip › ejph-2026-01-om-0092-File002.docx]

| **Supplementary table 1. Corresponding population census year and FDEP version applied to each year of mortality data** | | | | | | | | | | | | | | | | | | | | |
| --- | --- | --- | --- | --- | --- | --- | --- | --- | --- | --- | --- | --- | --- | --- | --- | --- | --- | --- | --- | --- |
| **Mortality data year** | | | | | | | | | | | | | | | | | | | | |
| 2000 | 2001 | 2002 | 2003 | 2004 | 2005 | 2006 | 2007 | 2008 | 2009 | 2010 | 2012 | 2013 | 2014 | 2015 | 2016 | 2017 | 2018 | 2019 | 2020 | 2021 |
| **Population census data year** | | | | | | | | | | | | | | | | | | | | |
| 1999 | 1999 | 1999 | 2006 | 2006 | 2006 | 2006 | 2006 | 2006 | 2011 | 2011 | 2011 | 2011 | 2016 | 2016 | 2016 | 2016 | 2016 | 2021 | 2021 | 2021 |
| **FDEP version year** | | | | | | | | | | | | | | | | | | | | |
| 1999 | 1999 | 1999 | 2009 | 2009 | 2009 | 2009 | 2009 | 2009 | 2009 | 2009 | 2009 | 2009 | 2015 | 2015 | 2015 | 2015 | 2015 | 2015 | 2015 | 2015 |

Note: Mortality data from 2011 was not included in our study due to missing region of birth.
