# Supplementary material for: Heterogeneous and dynamic lung cancer mortality among immigrants relative to native-born populations in France, 2000–2021
Source: Eur J Public Health. 2026 Jul 30;36(4):ckag134. doi: 10.1093/eurpub/ckag134 (PMC13424438; doi:10.1093/eurpub/ckag134)
Supplement: ckag134_Supplementary_Data [file ckag134_supplementary_data.zip › ejph-2026-01-om-0092-File005.docx]

| **Supplementary Table 4a. Mortality rate ratios (MRR) of lung cancer by social deprivation, sex and region of birth among women for 2000-2010 and 2012-2021** | | | | | | | | | | | | | | | | | | | | |
| --- | --- | --- | --- | --- | --- | --- | --- | --- | --- | --- | --- | --- | --- | --- | --- | --- | --- | --- | --- | --- |
|  | **Quintile 1 (lowest deprivation)** | | |  | **Quintile 2** | | |  | **Quintile 3** | | |  | **Quintile 4** | | |  | **Quintile 5 (highest deprivation)** | | |  |
| **Region of birth** | **ASMR (95% CI)** |  | **MRR (95% CI)** |  | **ASMR (95% CI)** |  | **MRR (95% CI)** |  | **ASMR (95% CI)** |  | **MRR (95% CI)** |  | **ASMR (95% CI)** |  | **MRR (95% CI)** |  | **ASMR (95% CI)** |  | **MRR (95% CI)** |  |
| **2000-2010** | | | | | | | | | | | | | | | | | | | | |
| **France** | 30.9 (30.3-31.5) |  | 1 (ref) |  | 27.9 (27.3-28.4) |  | 1 (ref) |  | 28.3 (27.8-28.9) |  | 1 (ref) |  | 27.1 (26.6-27.6) |  | 1 (ref) |  | 26.8 (26.3-27.3) |  | 1 (ref) |  |
| **All foreign-born** | 27.7 (26.5-29.1) |  | 0.88 (0.76-1.02) |  | 25.9 (24.6-27.3) |  | 0.94 (0.82-1.10) |  | 26.8 (25.4-28.3) |  | 0.93 (0.79-1.09) |  | 25.4 (24.1-26.9) |  | 0.91 (0.78-1.07) |  | 24.3 (23.1-25.7) |  | 0.85 (0.67-1.08) |  |
| **Southern Europe** | 20.9 (18.7-23.2) |  | 0.65 (0.54-0.80) |  | 19.7 (17.5-22.0) |  | 0.74 (0.59-0.93) |  | 20.2 (18.0-22.7) |  | 0.69 (0.55-0.87) |  | 20.5 (18.3-22.9) |  | 0.75 (0.60-0.93) |  | 20.7 (18.5-23.0) |  | 0.76 (0.57-1.00) |  |
| **Other European** | 39.0 (35.6-42.6) |  | 1.18 (0.98-1.42) |  | 38.5 (34.3-43.0) |  | 1.30 (1.03-1.63) |  | 42.5 (38.1-47.2) |  | 1.40 (1.12-1.75) |  | 38.0 (34.0-42.4) |  | 1.26 (1.01-1.57) |  | 40.6 (36.6-44.8) |  | 1.28 (0.98-1.67) |  |
| **Maghreb** | 22.9 (21.1-24.9) |  | 0.72 (0.60-0.87) |  | 24.1 (22.2-26.1) |  | 0.86 (0.70-1.07) |  | 24.4 (22.3-26.5) |  | 0.81 (0.66-1.01) |  | 21.7 (19.7-24.0) |  | 0.77 (0.62-0.95) |  | 18.2 (16.3-20.2) |  | 0.59 (0.45-0.78) |  |
| **Sub-Saharan Africa** | 32.9 (26.7-40.1) |  | 0.92 (0.72-1.17) |  | 30.9 (23.8-39.3) |  | 1.04 (0.78-1.38) |  | 28.3 (20.9-37.2) |  | 0.86 (0.80-1.49) |  | 31.0 (22.5-41.2) |  | 1.02 (0.74-1.38) |  | 26.1 (17.9-36.1) |  | 0.73 (0.50-1.07) |  |
| **Türkiye and Middle East** | 46.9 (36.5-59.2) |  | - |  | 40.1 (28.0-55.4) |  | - |  | 40.8 (27.4-58.3) |  | - |  | 25.5 (15.5-39.1) |  | - |  | 31.1 (20.0-45.4) |  | - |  |
| **Asia** | 35.1 (29.7-41.1) |  | 1.06 (0.85-1.33) |  | 32.1 (25.8-39.5) |  | 1.05 (0.79-1.39) |  | 36.6 (28.3-46.5) |  | 1.10 (0.80-1.49) |  | 44.5 (34.6-56.2) |  | 1.35 (0.99-1.81) |  | 44.0 (34.3-55.5) |  | 1.31 (0.93-1.83) |  |
| **Oceania/America** | 32.2 (23.7-42.6) |  | - |  | 24.2 (14.1-38.5) |  | - |  | 28.3 (16.0-45.3) |  | - |  | 29.8 (17.2-47.5) |  | - |  | 38.1 (21.4-61.9) |  | - |  |
| **2012-2021** | | | | | | | | | | | | | | | | | | | | |
| **France** | 35.8 (35.2-36.4) |  | 1 (ref) |  | 35.6 (35.0-36.1) |  | 1 (ref) |  | 36.4 (35.8-37.0) |  | 1 (ref) |  | 37.4 (36.9-38.0) |  | 1 (ref) |  | 39.8 (39.2-40.5) |  | 1 (ref) |  |
| **All foreign-born** | 28.5 (27.3-29.7) |  | 0.82 (0.72-0.92) |  | 27.9 (26.6-29.3) |  | 0.80 (0.68-0.94) |  | 27.4 (26.1-28.7) |  | 0.74 (0.64-0.85) |  | 30.7 (29.2-32.1) |  | 0.79 (0.65-0.96) |  | 26.4 (25.1-27.6) |  | 0.72 (0.55-0.93) |  |
| **Southern Europe** | 23.1 (20.8-25.6) |  | 0.69 (0.56-0.86) |  | 25.4 (22.8-28.1) |  | 0.73 (0.59-0.90) |  | 25.8 (23.2-28.7) |  | 0.72 (0.60-0.85) |  | 26.5 (23.6-29.6) |  | 0.72 (0.56-0.93) |  | 24.1 (21.4-26.9) |  | 0.65 (0.49-0.85) |  |
| **Other European** | 35.0 (31.9-38.3) |  | 0.95 (0.77-1.16) |  | 38.8 (34.8-43.0) |  | 1.08 (0.88-1.32) |  | 38.2 (34.5-42.1) |  | 1.00 (0.84-1.19) |  | 46.8 (42.7-51.2) |  | 1.15 (0.90-1.46) |  | 46.8 (42.6-51.2) |  | 1.12 (0.87-1.46) |  |
| **Maghreb** | 27.2 (25.3-29.1) |  | 0.79 (0.65-0.95) |  | 25.1 (23.2-27.1) |  | 0.71 (0.58-0.86) |  | 25.5 (23.6-27.4) |  | 0.68 (0.58-0.80) |  | 27.3 (25.3-29.6) |  | 0.66 (0.52-0.84) |  | 23.3 (21.5-25.1) |  | 0.59 (0.46-0.77) |  |
| **Sub-Saharan Africa** | 29.6 (25.1-34.5) |  | 0.81 (0.64-1.02) |  | 29.0 (23.8-34.8) |  | 0.78 (0.61-0.99) |  | 24.6 (19.8-30.1) |  | 0.63 (0.50-0.80) |  | 29.2 (23.4-35.7) |  | 0.72 (0.53-0.96) |  | 23.0 (17.8-29.1) |  | 0.54 (0.39-0.74) |  |
| **Türkiye and Middle East** | 45.3 (35.5-56.8) |  | - |  | 39.1 (27.7-53.5) |  | - |  | 17.3 (11.1-25.5) |  | - |  | 23.5 (15.5-33.9) |  | - |  | 19.4 (13.0-27.4) |  | - |  |
| **Asia** | 28.3 (24.3-32.7) |  | 0.79 (0.63-1.00) |  | 25.2 (20.3-30.9) |  | 0.67 (0.51-0.87) |  | 24.3 (19.2-30.3) |  | 0.63 (0.48-0.81) |  | 32.4 (25.5-40.4) |  | 0.78 (0.56-1.07) |  | 26.9 (21.0-33.8) |  | 0.67 (0.48-0.93) |  |
| **Oceania/America** | 29.4 (23.2-36.7) |  | - |  | 36.2 (25.5-49.5) |  | - |  | 35.4 (24.4-49.2) |  | - |  | 40.6 (27.6-57.2) |  | - |  | 19.6 (10.9-31.7) |  | - |  |

**Note**: Models adjusted on region of birth and 10-year age group. Cells marked “-” indicate censored region of birth groups due to low death count.
