# Supplementary material for: Heterogeneous and dynamic lung cancer mortality among immigrants relative to native-born populations in France, 2000–2021
Source: Eur J Public Health. 2026 Jul 30;36(4):ckag134. doi: 10.1093/eurpub/ckag134 (PMC13424438; doi:10.1093/eurpub/ckag134)
Supplement: ckag134_Supplementary_Data [file ckag134_supplementary_data.zip › ejph-2026-01-om-0092-File003.docx]

| **Supplementary table 2. Classification of countries in region of birth groups.** | |
| --- | --- |
| **Region group** | **Countries included** |
| **Southern Europe** | Andorra, Gibraltar, Italy, Monaco, Montserrat, Portugal, San Marino, Spain, Vatican City |
| **Other European** | Albania, Armenia, Austria, Azerbaijan, Belarus, Belgium, Bosnia and Herzegovina, Bouvet Island, Bulgaria, Cyprus, Croatia, Czechia, Denmark, Estonia, Faroe Islands, Finland, Georgia, Germany, Greece, Guernsey, Hungary, Iceland, Ireland, Isle of Man, Jersey, Kosovo, Latvia, Liechtenstein, Lithuania, Luxembourg, North Macedonia, Malta, Moldova, Montenegro, Norway, Netherlands, Poland, Romania, Russia, Serbia, Slovakia, Slovenia, Svalbard and Jan Mayen, Sweden, Switzerland, United Kingdom, Ukraine |
| **Maghreb** | Algeria, Morocco, Tunisia, Western Sahara |
| **Sub-Saharan Africa** | Angola, Benin, Botswana, British Indian Ocean Territory, Burkina Faso, Burundi, Cabo Verbe, Cameroon, Central African Republic, Chad, Comoros, Democratic Republic of the Congo, Republic of the Congo, Côte d'Ivoire, Djibouti, Egypt, Eritrea, Eswatini, Ethiopia, Equitorial Guinea, Gabon, Gambia, Ghana, Guinea, Guinea-Bissau, Kenya, Lesotho, Liberia, Libya, Madagascar, Malawi, Mali, Mauritania, Mauritius, Mozambique, Namibia, Niger, Nigeria, Rwanda, Saint Helena, Ascension and Tristan da Cunha, Sao Tome and Principe, Senegal, Seychelles, Sierra Leone, Somalia, South Africa, South Sudan, Sudan, Tanzania, Togo, Uganda, Zambia, Zimbabwe |
| **Türkiye and Middle East** | Bahrain, Iran, Iraq, Israel, Jordan, Kuwait, Lebanon, Oman, Palestine, Qatar, Saudi Arabia, Syria, Türkiye, United Arab Emirates, Yemen |
| **Asia** | Afghanistan, Bangladesh, Bhutan, Brunei, Cambodia, China, Democratic Republic of Korea, Republic of Korea, Hong-Kong, India, Indonesia, Japan, Kazakhstan, Kyrgyzstan, Laos, Macao, Malaysia, Maldives, Mongolia, Myanmar, Nepal, Pakistan, Philippines, Singapore, Sri Lanka, Tajikistan, Taiwan, Thailand, Timor-Leste, Turkmenistan, Uzbekistan, Viet Nam |
| **Oceania/America** | Anguilla, Antigua and Barbuda, Argentina, Aruba, Australia, Bahamas, Barbados, Belize, Bermuda, Bolivia, Bonaire, Sint Eustatius and Saba, Brazil, Cayman Islands, Canada, Chile, Christmas Island, Cocos Islands, Colombia, Cook Islands, Costa Rica, Cuba, Curaçao, Dominica, Dominican Republic, El Salvador, Ecuador, Falkland Islands, Fiji, Greenland, Grenada, Guadeloupe, Guam, Guatemala, Guyana, Haiti, Heard Island and McDonald Islands, Honduras, Jamaica, Kiribati, Northern Mariana Islands, Marshall Islands, Mexico, Micronesia, Nauru, Nicaragua, Niue, Norfolk Island, New Zealand, Palau, Panama, Papua New Guinea, Paraguay, Peru, Pitcairn, Puerto Rico, Saint Kitts and Nevis, Saint Lucia, Saint Martin, Saint Vincent and the Grenadines, Solomon Islands, Samoa, South Georgia and the South Sandwich Islands, Tokelau, Tonga, Trinidad and Tobago, Turks and Caicos Islands, Tuvalu, United States of America, Uruguay, Vanuatu, Venezuela, Virgin Islands |
