# Supplementary material for: Heterogeneous and dynamic lung cancer mortality among immigrants relative to native-born populations in France, 2000–2021
Source: Eur J Public Health. 2026 Jul 30;36(4):ckag134. doi: 10.1093/eurpub/ckag134 (PMC13424438; doi:10.1093/eurpub/ckag134)
Supplement: ckag134_Supplementary_Data [file ckag134_supplementary_data.zip › ejph-2026-01-om-0092-File006.docx]

| **Supplementary Table 4b. Mortality rate ratios (MRR) of lung cancer by social deprivation, sex and region of birth among men for 2000-2010 and 2012-2021.** | | | | | | | | | | | | | | | | | | | | | | | | | | | | | | | | |
| --- | --- | --- | --- | --- | --- | --- | --- | --- | --- | --- | --- | --- | --- | --- | --- | --- | --- | --- | --- | --- | --- | --- | --- | --- | --- | --- | --- | --- | --- | --- | --- | --- |
|  | **Quintile 1 (lowest deprivation)** | | | |  | | **Quintile 2** | | | | |  | | **Quintile 3** | | | | | |  | | **Quintile 4** | | | | |  | **Quintile 5 (highest deprivation)** | | | |  |
| **Region of birth** | **ASMR (95% CI)** |  | | **MRR (95% CI)** | |  | | **ASMR (95% CI)** |  | | **MRR (95% CI)** | |  | | **ASMR (95% CI)** | |  | | **MRR (95% CI)** | |  | | **ASMR (95% CI)** |  | | **MRR (95% CI)** |  | **ASMR (95% CI)** |  | | **MRR (95% CI)** |  |
| **2000-2010** | | | | | | | | | | | | | | | | | | | | | | | | | | | | | | | | |
| **France** | 113.0 (111.7-114.4) | | 1 (ref) | |  | | 124.8 (123.4-126.1) | | | 1 (ref) | |  | | 130.6 (129.4-131.9) | |  | | 1 (ref) | |  | | 133.4 (132.2-134.7) | | | 1 (ref) | |  | 150.0 (148.6-151.3) | | 1 (ref) | |  |
| **All foreign-born** | 111.9 (109.1-114.8) | | 1.00 (0.90-1.10) | |  | | 126.5 (123.4-129.7) | | | 0.98 (0.84-1.13) | |  | | 136.5 (133.1-140.0) | |  | | 1.00 (0.85-1.16) | |  | | 136.6 (133.2-140.1) | | | 0.98 (0.85-1.14) | |  | 143.1 (139.8-146.4) | | 0.97 (0.78-1.19) | |  |
| **Southern Europe** | 121.6 (115.6-127.8) | | 1.08 (0.95-1.22) | |  | | 135.5 (129.5-141.7) | | | 1.04 (0.90-1.20) | |  | | 142.0 (135.7-148.5) | |  | | 1.04 (0.87-1.23) | |  | | 149.2 (142.9-155.7) | | | 1.07 (0.93-1.23) | |  | 158.1 (151.8-164.6) | | 1.07 (0.89-1.29) | |  |
| **Other European** | 110.6 (103.6-118.0) | | 0.99 (0.87-1.13) | |  | | 126.5 (117.6-135.9) | | | 0.98 (0.84-1.14) | |  | | 141.4 (132.0-151.2) | |  | | 1.00 (0.84-1.20) | |  | | 134.7 (126.0-143.7) | | | 0.97 (0.83-1.13) | |  | 157.8 (148.9-167.1) | | 1.05 (0.86-1.27) | |  |
| **Maghreb** | 118.4 (113.9-122.9) | | 1.08 (0.95-1.21) | |  | | 128.9 (124.3-133.7) | | | 0.99 (0.86-1.14) | |  | | 135.9 (130.9-141.1) | |  | | 1.00 (0.85-1.19) | |  | | 135.4 (130.1-140.8) | | | 0.99 (0.86-1.14) | |  | 138.7 (133.8-143.8) | | 0.91 (0.76-1.09) | |  |
| **Sub-Saharan Africa** | 83.1 (72.8-94.3) |  | | 0.76 (0.65-0.88) | |  | | 78.0 (65.2-92.1) |  | | 0.62 (0.52-0.76) | |  | | 90.1 (73.8-108.1) | |  | | 0.61 (0.49-0.77) | |  | | 95.9 (78.4-115.5) |  | | 0.61 (0.50-0.75) |  | 83.2 (67.4-100.7) |  | | 0.48 (0.37-0.61) |  |
| **Türkiye and Middle East** | 114.4 (97.0-134.0) |  | | 0.95 (0.78-1.15) | |  | | 143.9 (118.6-172.6) | | | 1.05 (0.84-1.30) | |  | | 141.7 (114.9-172.3) | |  | | 0.95 (0.75-1.21) | |  | | 123.6 (97.5-153.2) |  | | 0.82 (0.65-1.02) |  | 124.1 (99.9-151.0) |  | | 0.73 (0.57-0.93) |  |
| **Asia** | 69.4 (60.4-79.1) |  | | 0.60 (0.50-0.71) | |  | | 81.1 (69.0-94.7) |  | | 0.59 (0.48-0.72) | |  | | 118.4 (100.0-139.1) | |  | | 0.74 (0.59-0.93) | |  | | 87.3 (71.2-105.8) |  | | 0.60 (0.48-0.75) |  | 93.3 (75.6-113.4) |  | | 0.53 (0.41-0.68) |  |
| **Oceania/America** | 64.9 (50.7-81.6) |  | | - | |  | | 71.3 (49.7-98.4) |  | | - | |  | | 76.9 (51.7-109.5) | |  | | - | |  | | 77.6 (51.1-112.1) |  | | - |  | 81.4 (52.6-119.3) |  | | - |  |
| **2012-2021** | | | | | | | | | | | | | | | | | | | | | | | | | | | | | | | | |
| **France** | 87.4 (86.3-88.5) |  | | 1 (ref) | |  | | 97.7 (96.7-98.8) |  | | 1 (ref) | |  | | 104.7 (103.6-105.8) | |  | | 1 (ref) | |  | | 112.5 (111.5-113.6) | | | 1 (ref) |  | 126.9 (125.6-128.1) | | | 1 (ref) |  |
| **All foreign-born** | 82.6 (80.5-84.8) |  | | 0.94 (0.92-0.97) | |  | | 89.6 (87.1-92.1) |  | | 0.92 (0.88-0.97) | |  | | 90.8 (88.4-93.3) | |  | | 0.88 (0.81-0.94) | |  | | 103.4 (100.7-106.1) | | | 0.93 (0.82-1.05) |  | 101.6 (99.2-104.1) |  | | 0.79 (0.67-0.94) |  |
| **Southern Europe** | 94.1 (89.0-99.3) |  | | 1.12 (0.92-1.37) | |  | | 100.4 (95.1-105.9) |  | | 1.04 (0.98-1.09) | |  | | 104.2 (98.8-109.9) | |  | | 1.04 (0.94-1.14) | |  | | 119.2 (113.2-125.4) | | | 1.11 (0.99-1.24) |  | 121.0 (115.1-127.0) | | | 0.99 (0.85-1.16) |  |
| **Other European** | 82.3 (76.6-88.4) |  | | 0.95 (0.78-1.16) | |  | | 92.6 (85.4-100.3) |  | | 0.95 (0.88-1.03) | |  | | 91.2 (84.6-98.2) | |  | | 0.89 (0.79-0.99) | |  | | 101.1 (94.4-108.2) |  | | 0.91 (0.80-1.03) |  | 114.1 (106.8-121.7) | | | 0.90 (0.77-1.06) |  |
| **Maghreb** | 92.1 (88.6-95.7) |  | | 1.15 (0.95-1.40) | |  | | 94.3 (90.6-98.2) |  | | 0.97 (0.93-1.00) | |  | | 92.7 (89.1-96.3) | |  | | 0.90 (0.82-0.98) | |  | | 108.2 (104.0-112.4) | | | 0.97 (0.87-1.08) |  | 104.3 (100.7-108.0) | | | 0.82 (0.71-0.95) |  |
| **Sub-Saharan Africa** | 48.3 (42.7-54.3) |  | | 0.62 (0.50-0.77) | |  | | 48.3 (41.8-55.4) |  | | 0.56 (0.50-0.63) | |  | | 55.9 (48.5-64.1) | |  | | 0.57 (0.49-0.66) | |  | | 64.4 (54.6-75.2) |  | | 0.54 (0.45-0.64) |  | 46.9 (40.2-54.3) |  | | 0.36 (0.30-0.44) |  |
| **Türkiye and Middle East** | 102.8 (88.7-118.4) |  | | 1.22 (0.98-1.51) | |  | | 99.9 (81.4-121.1) |  | | 0.88 (0.73-1.04) | |  | | 98.3 (81.9-116.8) | |  | | 0.86 (0.72-1.02) | |  | | 95.8 (80.3-113.1) |  | | 0.79 (0.66-0.95) |  | 114.8 (99.6-131.3) |  | | 0.80 (0.66-0.97) |  |
| **Asia** | 47.7 (41.9-54.1) |  | | 0.59 (0.47-0.73) | |  | | 57.3 (49.1-66.3) |  | | 0.57 (0.49-0.65) | |  | | 52.2 (43.8-61.7) | |  | | 0.50 (0.42-0.50) | |  | | 55.7 (45.9-66.8) |  | | 0.49 (0.40-0.59) |  | 49.4 (41.0-58.9) |  | | 0.37 (0.29-0.46) |  |
| **Oceania/America** | 39.7 (30.6-50.4) |  | | - | |  | | 42.6 (29.1-59.7) |  | | - | |  | | 61.5 (43.5-83.9) | |  | | - | |  | | 60.6 (41.5-84.8) |  | | - |  | 62.7 (41.2-90.5) |  | | - |  |

**Note**: Models adjusted on region of birth and 10-year age group. Cells marked “-” indicate censored region of birth groups due to low death count.
